# Objectively-measured step cadence and walking patterns in a rural African setting: a cross-sectional analysis

**DOI:** 10.1186/s13104-022-06045-9

**Published:** 2022-05-04

**Authors:** Ian Cook

**Affiliations:** Physical Activity Epidemiology Laboratory (EDST), Faculty of Humanities, University of Limpopo (Turfloop Campus), University Road, Mankweng-B, 0727 Limpopo Province South Africa

**Keywords:** Ambulation, Pedometry, Movement monitor, Measurement

## Abstract

**Objectives:**

To investigate free-living, accelerometry-derived step cadence and walking strategy parameters in 263 adult women (19–56 years) within a rural African setting. Participants were categorised into weight groups: Under-to-Normal Weight (UW/NW: < 25 kg/m^2^), Overweight-to-Obese (OW/OB: ≥ 25 kg/m^2^). From the minute-by-minute uni-axial accelerometry data, outcomes describing physical activity intensity, step volume, step cadence and step bouts were extracted. In addition, walking pattern parameters for step bout length and step cadence were determined.

**Results:**

Average step volume was 13,568 steps/day, and > 85% of participants were classified as active-to-highly-active. Overall, ≈ 45% of daily steps was accumulated in the low-to-moderate intensity range. Peak cadence indices were higher in the UW/NW group (*p* ≤ 0.0112). For both groups, 75% of steps were accumulated in bouts > 15 min, and 95% of bouts were accumulated at 1–39 steps/min. The UW/NW group employed a more varied step cadence, and higher cadences contributed more to step accumulation than the OW/OB group (*p* ≤ 0.05). There were no significant group differences in bout length strategy parameters (*p* ≥ 0.0861). Despite no difference between the weight groups in step volume, there were differences in some step cadence indices which reflect higher step intensities, and in cadence strategies chosen to accumulate steps.

**Supplementary Information:**

The online version contains supplementary material available at 10.1186/s13104-022-06045-9.

## Introduction

Active transport, in particular walking, as part of a rural, subsistence lifestyle contributes significantly to the total physical activity volume in non-industrialised settings [1]. Rural women in South Africa can spend up to 224 min/day on housework, and collecting wood and water [2]. In a rural setting the time spent in subsistence activities is likely reflected in high physical activity volumes [3, 4]. However, these analyses provided no insight as to how these steps are accumulated, specifically in terms of walking intensity (step cadence) [5] and walking patterns (step cadence and bouts) [6, 7]. Given the link between step indices and health [8, 9], the exploration of step indices is warranted, especially in rural settings where step cadence data is sparse. The strategy chosen to accumulate steps in this rural setting is likely influenced by health and socio-economic factors [10]. To date, there are no South African studies reporting free-living step cadence and step bout indices, and how these step indices are expressed in walking patterns. Hence, the objective of this cross-sectional analysis is to explore the step cadence and step bout patterns in a group of rural African women, and expand the findings of earlier objectively-measured physical activity data [3, 4].

## Main text

### Methods

The data for this analysis has been reported in detail elsewhere [4]. Rural, adult females resident in the Dikgale Health and Demographic Surveillance System site (DHDSS) [11], were conveniently recruited during 2003–2004 (n = 263). The participants generally performed subsistence tasks (housework, fetching wood and water, walking).

#### Data collection and initial data reduction

In short, anthropometric and questionnaire data (health, socio-economic) were collected by trained field workers [4]. Body Mass Index (BMI) was calculated from stature (m) and body mass (kg) and classified as underweight-to-normal weight (UW/NW, < 25 kg/m^2^) and overweight-to-obese (OW/OB, ≥ 25 kg/m^2^). A Physical Activity Index (PAI) was calculated from four socio-economic factors [3]. The presence of disease was defined as diagnosed with and/or on medication for hypertension, diabetes mellitus, heart disease and/or hypercholesterolemia.

Thereafter, participants were asked to wear uni-axial accelerometers (MTI model AM-7164-2.2, Actigraph, LLC, Pensacola, FL, USA) affixed to the waist for seven days. The minute-by-minute data were downloaded from the accelerometers onto a personal computer (Windows Operating System) via an interface unit, for further analysis using specialized software (MAH/UFFE Analyzer version 1.9.0.3; http://www.mrc-epid.cam.ac.uk/physical-activity-downloads/). The initial data reduction methodology is described in detail elsewhere [4].

In addition to minute-by-minute step counts, minute-by-minute acceleration counts were classified as physical activity volumes of sedentary, light, moderate and vigorous activities using previously defined cut-points (see Additional file 1) [12, 13].

#### Additional data reduction and walking pattern analysis

For this analysis individual, minute-by-minute data files (CSV) created with MAH/UFFE were batch-converted to individual Microsoft Excel files using a custom Microsoft Visual Basic macro. Thereafter, the data for non-valid days and non-wear time (identified in the initial data reduction) were removed for each individual, minute-by-minute Microsoft Excel file using a customized Microsoft Visual Basic macro. The required step-based, walking cadence and accelerometer count parameters (see Additional file 1) [5, 14–17] were extracted for each cleaned, individual minute-by-minute Microsoft Excel file using customized Microsoft Visual Basic macros. The resulting summary Microsoft Excel files were imported into appropriate statistical packages for further analysis.

From the extracted step bout frequency, bout duration and cadence data, additional walking pattern parameters (*G*: Gini index, S_2w_: within-subject variability) were estimated using non-binned data for each participant (see Additional file 1) [6, 7]. The within-subject variability of bout lengths and cadence was obtained by maximum likelihood estimation methods [18].

#### Statistical analysis

Descriptive statistics comprised means and one standard deviation (sd), median and interquartile range (iqr), and frequencies. Bivariate relationships were examined using linear regression. Relationships between categorical variables were examined through Fisher’s Exact Test. For continuous data, independent *t* tests examined differences between groups. Where required a non-parametric test was employed. A Two-way Analysis of Variance was conducted between BMI groups and the steps accumulated in the four accelerometer count bands. Post hoc multiple comparison tests (Sidak) were run between BMI groups for each accelerometer count band. Independent relationships between walking pattern parameters (*G*, S_2w_) and variables identified as significant during bivarate analyses were examined using forced multiple linear regression models. Model assumptions were examined [19]. Cumulative density plots for step accumulation were constructed for walking bouts, and bouted and unbouted cadence. Heat maps were constructed by bout length, bout frequency and bouted cadence step categories.

Data were analysed using appropriate statistical software (Stata/SE for Windows: Release 17.0 College Station, TX: StataCorp LP, 2021 and GraphPad Prism: version 8.3.0, GraphPad Software, La Jolla CA, 2019). Significance was set at *p* ≤ 0.05.

### Results

Other than age, anthropometric indices and one socio-demographic variable (*p* ≤ 0.0144), there were no significant differences between BMI groups across basic descriptive characteristics. There was a non-significant tendency for a lower subsistence score in the OW/OB group (*p* = 0.083) (Table 1).

**Table 1:** Descriptive statistics of female participants by weight status

	Combined (n = 263)	Under-Normal weight (n = 122)	Overweight-Obese (n = 141)	*p*-value ^c^
Age (years)	35.1 (10.5)	33.3 (11.4)	36.7 (9.5)	**0.0096**
Age distribution (quartiles)^a^				
18.7–24.5 years	–	34.4 (42)	16.3 (23)	**0.0050**
24.6–36.1 years	–	23.8 (29)	26.2 (37)	
36.2–42.6 years	–	18.9 (23)	30.5 (43)	
42.7–56.2 years	–	23.0 (28)	27.0 (38)	
BMI (kg/m^2^)	26.8 (6.0)	21.8 (2.1)	31.2 (4.7)	** < 0.0001**
BMI distribution^a^				
Underweight (< 18.5 kg/m^2^)	3.0 (8)	–	–	
Normal weight (18.5–24.9 kg/m^2^)	43.3 (114)	–	–	
Overweight (25–29.9 kg/m^2^)	22.8 (60)	–	–	
Obese (30–34.9 kg/m^2^)	22.4 (59)	–	–	
Severe obesity (≥ 35 kg/m^2^)	8.4 (22)	–	–	
Waist circumference (cm)	82.6 (12.9)	72.5 (6.0)	91.2 (10.9)	** < 0.0001**
Disease present (Yes)^ab^	19.0 (50)	18.0 (22)	19.9 (28)	0.4144
Electricity inside the house (Yes)^a^	86.3 (227)	86.1 (105)	86.5 (122)	0.5273
Wood used for cooking purposes (Yes)^a^	86.7 (228)	89.3 (109)	84.4 (119)	0.1597
One or more persons in the household owns a motor vehicle (Yes)^a^	18.3 (48)	12.3 (15)	23.4 (33)	**0.0144**
Water supplied by tap in or around dwelling (Yes)^a^	60.1 (158)	59.0 (72)	61.0 (86)	0.4205
Physical Activity Index^a^				
Low subsistence level (low activity)	67.7 (178)	62.3 (76)	72.3 (102)	0.0830
Medium subsistence level (medium activity)	20.9 (55)	27.0 (33)	15.6 (22)	
High subsistence level (high activity)	11.4 (30)	10.7 (13)	12.1 (17)	

Unadjusted values are reported as mean(sd) except^a^ %(n)

^b^Disease: diagnosed with and/or on medication for hypertension, diabetes mellitus, heart disease and/or hypercholesterolemia

^c^significant difference, p < 0.05: Under-Normal weight (Body Mass Index: < 25 kg/m^2^) vs. Overweight-Obese (Body Mass Index: ≥ 25 kg/m2), continuous variables: independent t test; categorical variables: Fisher’s exact test

Walking volume indices (total daily steps and bouts, maximum bout length) were not significantly different between BMI groups (*p* ≥ 0.0629) (Table 2). Compared with OW/OB participants, UW/NW participants displayed significantly higher peak 1-min walking cadences, spent more time walking at cadences ≥ 100 steps/min, and accumulated more bouts and longer maximum bouts at ≥ 100 steps/min (*p* ≤ 0.0463). OW/OB participants spent less time in sedentary situations (0 steps/min), but more time in situations resulting in incidental steps (1–19 steps/min), than UW/NW participants (*p* ≤ 0.0112). There was a non-significant tendency for OW/OB participants to choose a more varied bout length (higher S_2w_) and longer walking bouts tended to contribute more to patterns of step accumulation (higher *G*), compared with UW/NW participants (*p* ≥ 0.0861). In contrast, in UW/NW participants, walking parameters (S_2w_, *G*) for bouted cadence were significantly higher compared with OW/OB participants (*p* ≤ 0.005). In other words, UW/NW participants displayed a more varied bouted cadence choice, and higher bouted cadences contributed more to the accumulation of steps, compared with OW/OB participants (Table 2).

**Table 2:** Descriptive statistics of ambulation indices by weight status

	Combined (n = 263)	Under-Normal weight (n = 122)	Overweight-Obese (n = 141)	*p*-value^d^
Number of days monitored				
All	5.5 (1.6)	5.4 (1.6)	5.4 (1.6)	0.5560
Weekdays	3.7 (1.2)	3.8 (1.2)	3.7 (1.2)	0.2037
Weekend days	1.7 (0.6)	1.7 (0.7)	1.7 (0.6)	0.4095
Average steps per day	13,568 (3571)	13,817 (3599)	13,353 (3545)	0.2937
Total walking bouts ≥ 1 min (bouts/day)	78 (17)	80 (17)	76 (17)	0.0629
Longest walking bout ≥ 1 min (minutes)	146 (70)	144 (66)	150 (69)	0.4672
Peak 1-min cadence (steps/min)	114 (13)	116 (13)	112 (12)	**0.0063**
Peak non-consecutive 30-min cadence (steps/min)	93 (14)	94 (14)	91 (13)	0.0564
Peak consecutive 30-min cadence (steps/min)	63 (16)	64 (16)	62 (16)	0.2932
Time in cadence band (min/day)^a^				
0 steps/min	273 (123)	289 (105)	248 (117)	**0.0006**
1–19 steps/min (Incidental movement)	332 (76)	324 (62)	341 (80)	**0.0112**
20–39 steps/min (Sporadic movement)	108 (50)	110 (46)	106 (52)	0.6449
40–59 steps/min (Purposeful steps)	47 (26)	47 (25)	47 (28)	0.8888
60–79 steps/min (Slow walking)	22 (11)	21 (10)	22 (12)	0.9948
80–99 steps/min (Medium walking)	19 (15)	20 (13)	19 (17)	0.8434
100–119 steps/min (Brisk walking)	17 (17)	19 (17)	13 (15)	**0.0096**
≥ 120 steps/min (Including all faster ambulation)	1 (3)	1 (4)	1 (2)	**0.0209**
Walking pattern^b^				
Bout length				
S_2w_	1.167 (0.116)	1.154 (0.115)	1.178 (0.115)	0.0894
*G*	0.589 (0.046)	0.584 (0.045)	0.594 (0.046)	0.0861
Cadence				
Bouted S_2w_	1.274 (0.099)	1.292 (0.083)	1.258 (0.108)	**0.0050**
Bouted *G*	0.631 (0.038)	0.638 (0.031)	0.625 (0.420)	**0.0040**
Unbouted S_2w_	1.394 (0.107)	1.400 (0.108)	1.389 (0.106)	0.3971
Unbouted *G*	0.675 (0.037)	0.677 (0.037)	0.673 (0.037)	0.4011
Bout characteristics for step cadence ≥ 100 steps/min^a^				
Total walking bouts ≥ 1 min (bouts/day^−1^)	7 (6)	8 (4)	5 (5)	**0.0179**
Maximum bout duration ≥ 1 min (minutes)	5.3 (5.8)	6.0 (5.5)	4.5 (5.7)	**0.0463**
Proportion of all walking bouts (%)				
≥ 1 min bout	7.7 (7.6)	8.4 (7.1)	7.1 (8.1)	0.0666
1 min bout	3.2 (3.3)	3.6 (2.9)	2.9 (3.9)	0.0960
2 min bout	1.5 (1.7)	1.6 (1.7)	1.3 (1.6)	0.0941
5 min bout	0.3 (0.6)	0.3 (0.6)	0.3 (0.6)	0.2617
≥ 10 min bout	0.3 (0.7)	0.3 (0.9)	0.2 (0.6)	0.1413

Unadjusted values are reported as mean (sd) except

^a^Median (iqr)

^b^S_2w_: within-subject distribution, *G*: Gini coefficient

^c^bouted: average cadence within a continuous bout of steps (bout ≥ 1 min), unbouted: minute-by-minute cadence

^d^significant difference, *p* < 0.05: Under-Normal weight (Body Mass Index: < 25 kg/m^2^) vs. Overweight-obese (Body Mass Index: ≥ 25 kg/m^2^), continuous variables: independent *t* test or Mann–Whitney *U* test; categorical variables: Fisher’s exact test

**Fig. 1:**
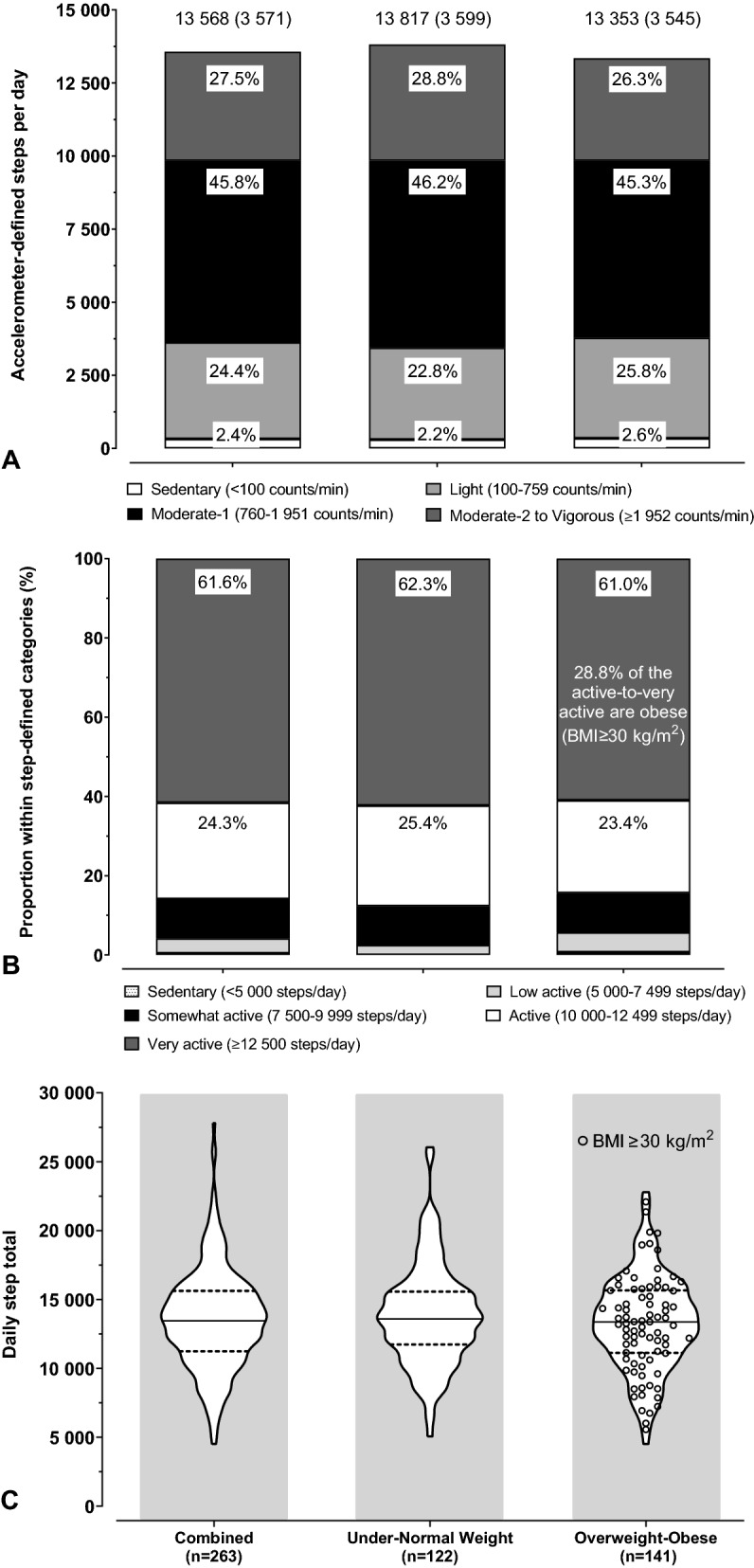
Accelerometry and pedometry indices across weight status. **A** Steps accumulated within accelerometer count bands; **B** Proportion of participants within step-defined categories; **C** Average daily step counts

For both BMI groups accumulated steps were relatively equally distributed in the Light and Moderate-2-to-Vigorous categories and ≈ 45% of daily steps were accumulated in the Moderate-1 accelerometer band (760–1951 counts/min) (Fig. 1A). There were no significant differences between the BMI groups for steps accumulated in the four accelerometer count bands (*p* ≥ 0.0911).

More than 85% of the participants were classified as active-to-very active, irrespective of BMI status (Fig. 1B). There was no association between BMI category and step-defined activity categories (*p* = 0.7958).

BMI groups did not differ for average daily steps (Fig. 1C, p = 0.2937) and there was no difference (*p* = 0.5633) in the average daily steps between overweight (BMI 25–29.9 kg/m^2^) and obese (BMI ≥ 30 kg/m^2^) participants (13 489 steps/day and 13 153 steps/day, respectively).

For both BMI groups, a greater percentage of total steps were accumulated in longer walking bouts (75% of total steps at > 15 min bout length), than shorter, more frequent bouts (see Additional file 2: Fig. S1 A).

The majority of the step accumulation occurs at the lower end of the bouted cadence spectrum (see Additional file 2: Fig. S1 B). Noticeably, in the UW/NW group, in the range of 50–90% of the percentage of total steps accumulated, steps were accumulated at higher bouted cadences.

When ignoring walking bouts, and only considering the minute-by-minute accumulation of steps (unbouted), a greater number of steps are accumulated at higher walking cadences (see Additional file 1: Fig. S1 C) compared with bouted cadence steps (see Additional file 1: Fig. S1 B). The difference between the BMI groups was not as marked as for the bouted cadence step accumulation pattern in Additional file 2: Fig. S1 B.

There was a general similarity in the distribution of walking bout length and frequency patterns across cadence bands (see Additional file 3: Fig. S2 A–C). Approximately 95% of walking bouts were accumulated in the lowest two walking cadences (1–39 steps/min), with approximately 82% of walking bouts accumulated in the lowest cadence (1–19 steps/min) primarily through fewer, longer bout lengths. Walking cadences ≥ 60 steps/min were associated with reductions in walking bout length and frequency, and the trend was consistent across BMI groups. Walking bouts were rare at cadences ≥ 100 steps/min with a frequency of ≤ 5 bouts over the monitoring period (see Additional file 3: Fig. S2 A–C).

In bivariate analyses, walking bout length S_2w_ and *G* were not significantly associated with age, BMI, disease presence, PAI or any of the components of PAI (*p* ≥ 0.063). Bouted cadence S_2_ and *G* were significantly and inversely associated only with BMI (*p* < 0.001). Age, presence of disease, availability of electricity in the dwelling and PAI were significantly associated with unbouted cadence S_2w_ and *G* (*p* ≤ 0.038). Hence, forced multiple linear regression models were run with age, presence of disease and availability of electricity or PAI as independent variables, and unbouted cadence S_2w_ and *G* as dependent variables. All model assumptions were met. Independent of age (*p* = 0.140) and the presence of disease (*p* = 0.054), availability of electricity in the dwelling was inversely and significantly associated with unbouted cadence S_2w_ (*p* = 0.040, *β* = − 0.0392) (Model: *p* = 0.0042, adjusted R^2^ = 0.0386). Similarly, independent of age (*p* = 0.132), the presence of disease (*p* = 0.049, *β* =  + 0.01149) and availability of electricity in the dwelling (*p* = 0.042, *β* = − 0.01344) were significantly associated with unbouted cadence *G* (Model: *p* = 0.0037, adjusted R^2^ = 0.0395). PAI was not significant in any model (*p* ≥ 0.061).

### Discussion

This analysis is novel in that, as far as the author is aware, this is the first step cadence and walking pattern analysis from a South African context, specifically a rural setting. The major findings are first that there was no difference between the weight groups in average daily step volumes. Second, there were significant differences between weight groups in some step cadence indices which suggest higher step intensities. Third, cadence strategies chosen to accumulate steps differed between weight groups and walking pattern parameters were significantly associated with anthopometric, health and socio-economic variables.

DHDSS females accumulate nearly 5000 uncensored steps/day more compared with females from a highly industrialised setting (NHANES), and accumulate a greater percentage of steps in the moderate-2-to-vigorous accelerometer band and a lower percentage of steps in the sedentary-to-light accelerometer band (+ 6% and − 7%, respectively) [17]. Interestingly, the percentage of steps accumulated in the moderate-1 accelerometer band is similar (DHDSS: 45.8% versus NHANES: 46.7%) [17]. Furthermore DHDSS females spend 61 min less time in the 1–19 steps/min cadence band and 62 min more time in the 20–119 steps/min cadence bands, compared with NHANES females [20]. Peak 1-min and 30-min cadences are 13% and 33% higher in DHDSS females, respectively [21].

The OW/OB group use a less varied choice of bouted step cadence during ambulation, and a more equal distribution of bouted step cadences determines step accumulation. A lower walking speed results in a lower energy cost [22] and a lower step cadence can reduce the perception of effort in obese individuals [23]. Choosing a slower speed, over a set distance lowers the relative effort and perception [22, 23] but increases walking bout length. Indeed, although not statistically significant (*p* ≤ 0.0861), OW/OB walking bout length parameters indicated a more varied choice of bout length, and longer walking bouts.

Not having electricity supplied into the dwelling, will likely result in an increase in physical movement patterns through manual activities and an increased reliance on collecting wood for cooking and heating purposes [3]. This could explain the more varied choice in unbouted cadence and higher unbouted cadences contributing to overall step volume. The presence of disease would require more regular visits to clinics and hospitals which are on average ≈ 5 km from rural homesteads [24–26]. Average self-report walking time to a clinic is 62.3 min, and assuming walking speeds of 2–4 km/hour [27], a 5 km trip would result in walking bouts of 60–150 min. This would likely result in higher step cadences contributing more to step accumulation.

In conclusion, this report suggests that rural African women, within a specific setting, accumulate high step volumes through choices in bout length and cadence patterns which are informed by anthopometric, health and socio-economic variables.

## Limitations

Due to the cross-sectional, convenience sampling in this study, the results cannot be readily generalized to the respective rural population from whence the participants were recruited.

## Supplementary Information

**Additional file 1: Definitions.docx.** Detailed definitions of accelerometer cut-points, and step-based and walking cadence parameters

**Additional file 2**: **Figure S1.** Cumulative distribution of total steps as a function of walking indices.** A**. Accumulation of steps by bout length; **B**. Accumulation of steps by bouted cadence; **C**. Accumulation of steps by unbouted cadence

**Additional file 3**: **Figure S2.** The distribution of walking bouts and bout frequency across bouted cadence categories.** A**. Full sample (112 774 walking bouts); **B**. UW/NW group (54 282 walking bouts); **C**. OW/OB group (58 492 walking bouts)

## Data Availability

The data analysed during the current study are not publicly available due to the original consent and ethics approval not containing approval from the participants for data sharing. Reasonable requests would be considered in consultation with the University of Limpopo Ethics Committee and the various community leaders.

## Abbreviations

DHDSS: Demographic and Health Surveillance System Site
BMI: Body Mass Index
*G*: Gini index
NW: Normal weight
OB: Obese
OW: Overweight
PAI: Physical Activity Index
UW: Under weight
S_2w_: Within-subject variability
