# Supplementary material for: Objectively-measured step cadence and walking patterns in a rural African setting: a cross-sectional analysis
Source: BMC Res Notes. 2022 May 4;15:155. doi: 10.1186/s13104-022-06045-9 (PMC9069760; doi:10.1186/s13104-022-06045-9)
Supplement: Supplementary file 1 — Additional file 1: Definitions.docx. Detailed definitions of accelerometer cut-points, and step-based and walking cadence parameters [file 13104_2022_6045_MOESM1_ESM.docx]

# Detailed definitions of accelerometer cut-points, and step-based and walking cadence parameters

- Minute-by-minute acceleration count cut-points: sedentary (0-99 counts/min), light (100-760 counts/min), moderate activity (moderate-1: 760-1951 counts/min, moderate-2: 1952-5724 counts/min) and vigorous activity (≥5725 counts/min) [12, 13]
- Average daily ambulatory activity (steps/day): Sedentary: <5000 steps/day, Low active: 5000-7499 steps/day, Somewhat active: 7500-9999 steps/day, Active: ≥10 000 steps/day, and Very active: ≥12 500 steps/day [14]
- Frequency of each bout duration, where bout duration ranges from 1 minute to ≥420 minutes with 1 minute increments
- Steps accumulated within each accelerometer count band: sedentary (0-99 counts/min), light (100-760 counts/min), moderate-1 activity (moderate-1: 760-1951 cts.min^-1^and moderate-2 to vigorous activity (≥1952 counts/min) [17]
- Steps accumulated within each walking bout, where bout duration ranges from 1 minute to ≥420 minutes with 1 minute increments
- Steps accumulated and time spent in each step cadence band (steps/min): 0 steps/min, 1-19 steps/min (Incidental movement), 20-39 steps/min (Sporadic movement), 40-59 steps/min (Purposeful steps), steps/min (Slow walking), 80-99 steps/min (Medium walking), 100-119 steps/min (Brisk walking), ≥120 steps/min (Including all faster ambulation) [5]
- Bouted Steps: Steps accumulated for each average cadence within a continuous bout of steps, where bout duration ranges from 1 minute to ≥420 minutes with 1 minute increments
- Unbouted Steps: Steps accumulated at each minute-by-minute step cadence
- Longest walking bout (minutes), irrespective of step cadence
- Peak 1-minute cadence (steps/min) [5]
- Peak consecutive and non-consecutive 30-minute cadence (steps/min) [5]
- Number of bouts for ≥1-, ≥10-, 1-, 2-, 5- and 10- minutes, and the maximum bout duration (minutes) for a step cadence ≥100 steps/min [5, 15, 16]
- *G* (Gini index) and S_2w_ (within-subject variability): A higher S_2w_ is indicative of a walking pattern utilizing a more varied choice of bout lengths or cadence. The Gini index (*G*) characterises how total steps are accumulated from different step bout lengths or cadences. *G* varies between 0 and 1. A high *G* (closer to 1) indicates that long walking bouts or higher cadences contribute more to the pattern of accumulation. A low *G* (closer to 0) indicates that the accumulation is more fragmented and that all walking bout lengths or cadences contribute equally to total steps [6, 7]
