# Supplementary figures and images for: Objectively-measured step cadence and walking patterns in a rural African setting: a cross-sectional analysis
Source: BMC Res Notes. 2022 May 4;15:155. doi: 10.1186/s13104-022-06045-9 (PMC9069760; doi:10.1186/s13104-022-06045-9)

A

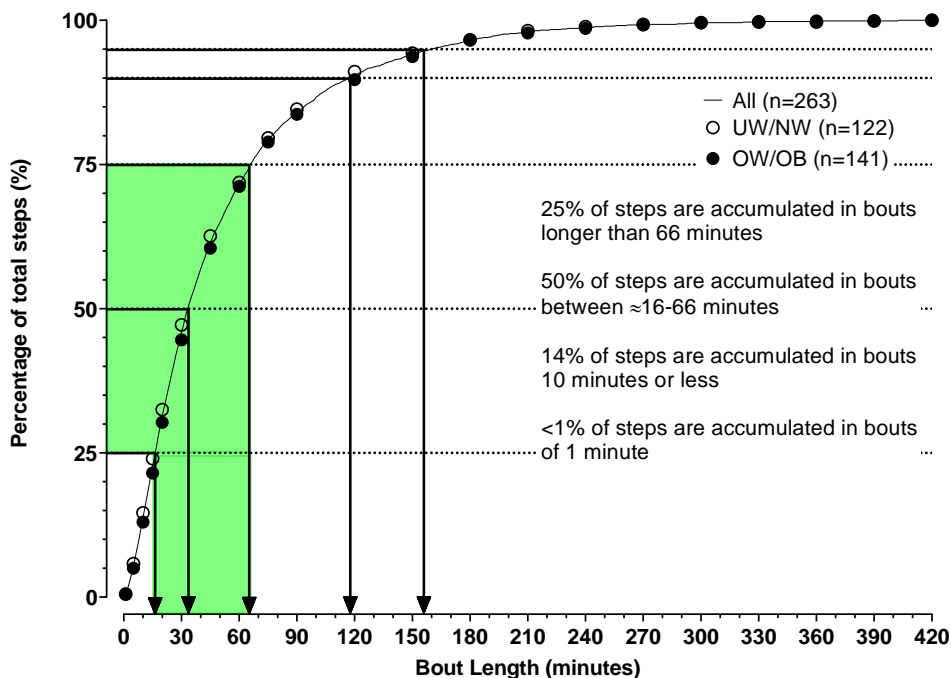

B

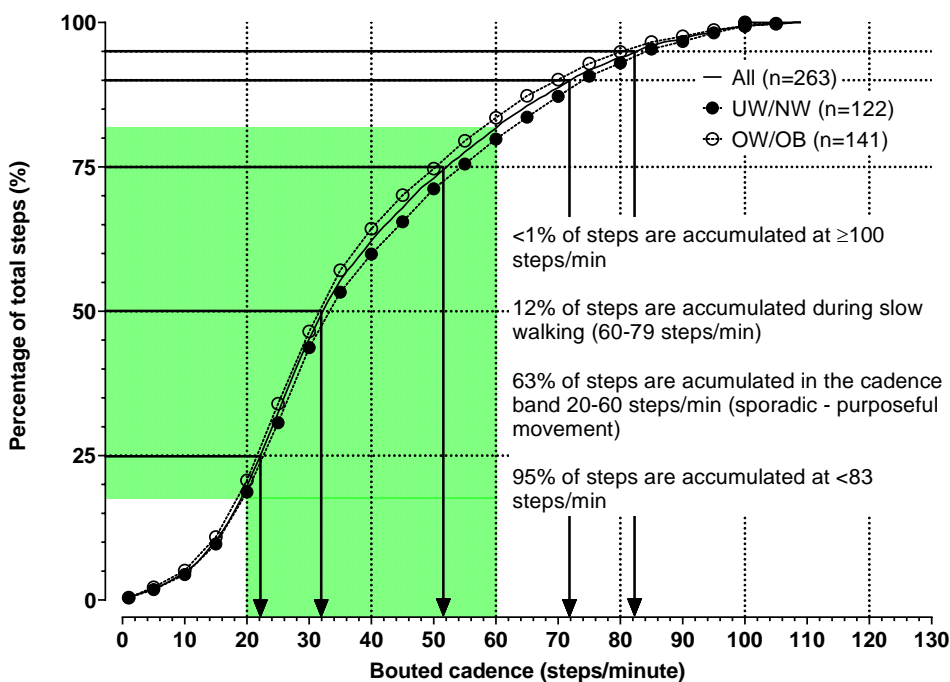

C

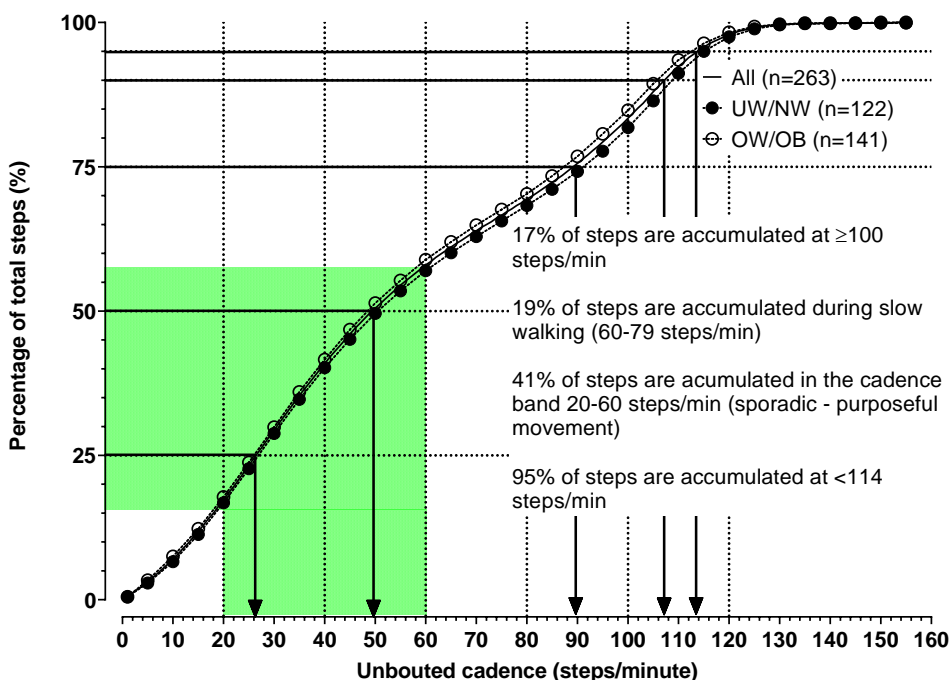

Supplement: Supplementary file 2 — Additional file 2: Figure S1. Cumulative distribution of total steps as a function of walking indices. A. Accumulation of steps by bout length; B. Accumulation of steps by bouted cadence; C. Accumulation of steps by unbouted cadence [file 13104_2022_6045_MOESM2_ESM.pdf]

A

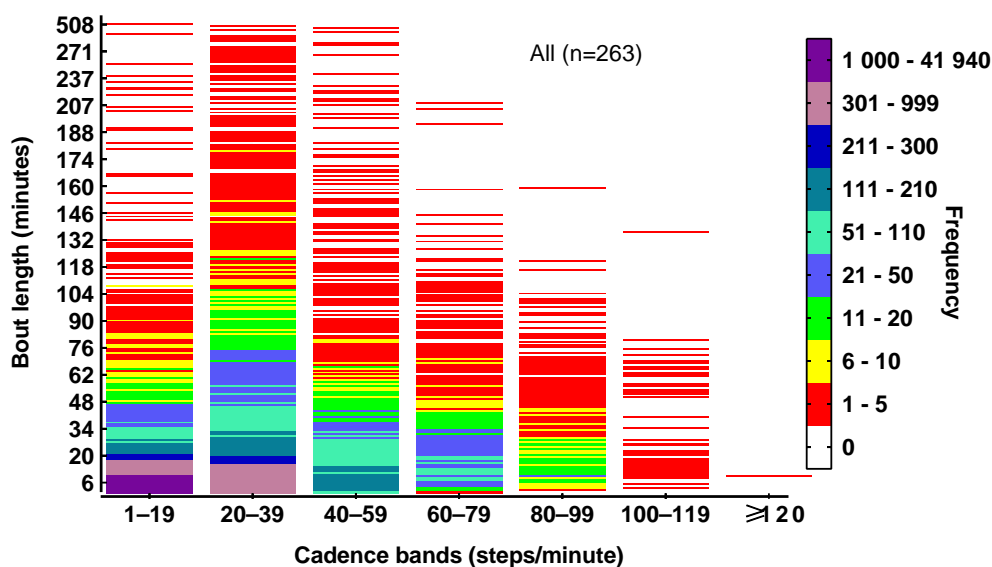

B

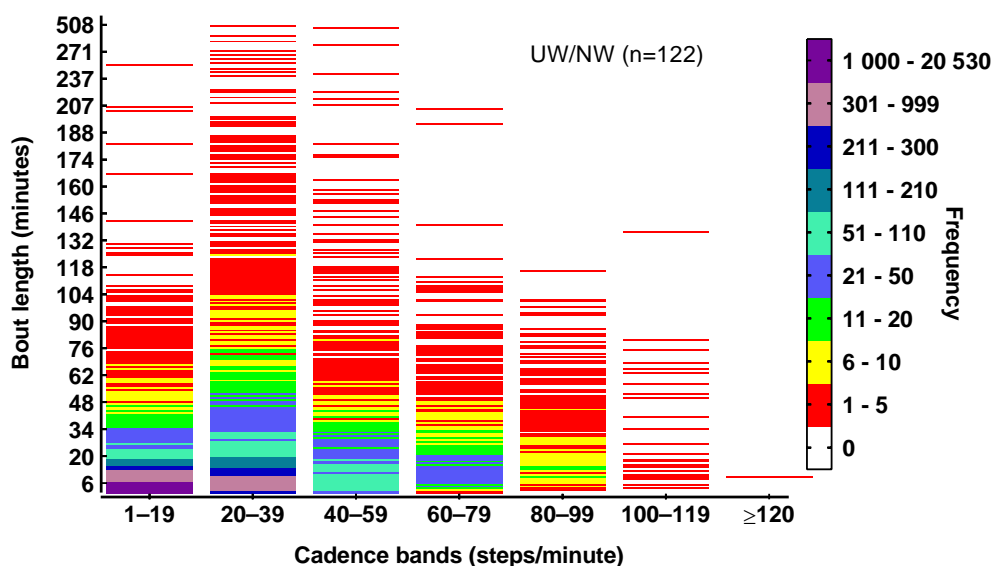

C

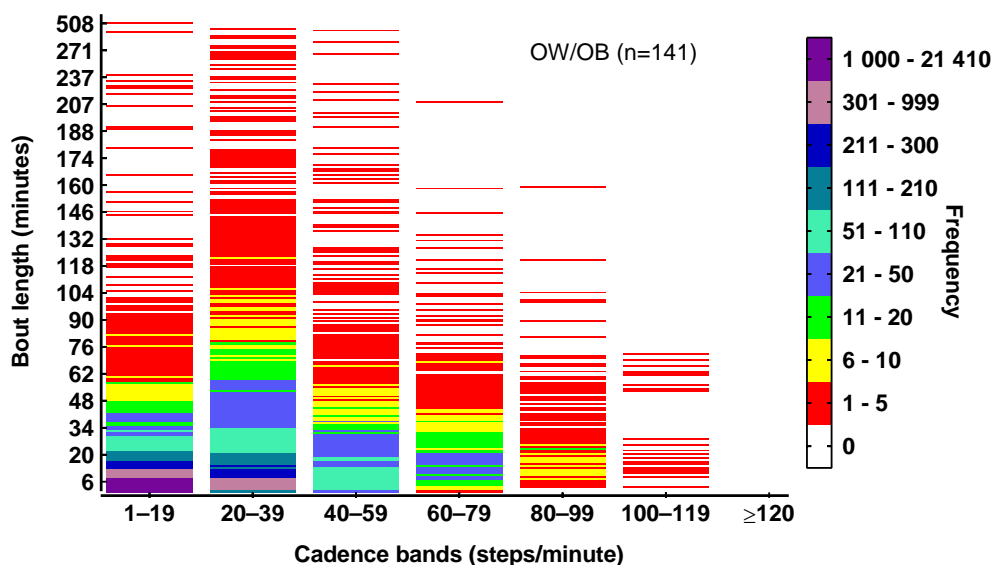

Supplement: Supplementary file 3 — Additional file 3: Figure S2. The distribution of walking bouts and bout frequency across bouted cadence categories. A. Full sample (112 774 walking bouts); B. UW/NW group (54 282 walking bouts); C. OW/OB group (58 492 walking bouts) [file 13104_2022_6045_MOESM3_ESM.pdf]
